# A Paradigm Shifting View of Intellectual Disability: A Near Normal Distribution of IQ in Fragile X Syndrome

**DOI:** 10.21203/rs.3.rs-2869313/v1

**Published:** 2023-05-05

**Authors:** Lauren M. Schmitt, Meredith Will, Rebecca Shaffer, Craig Erickson

**Affiliations:** Cincinnati Children’s Hospital Medical Center; Cincinnati Children’s Hospital Medical Center; Cincinnati Children’s Hospital Medical Center; Cincinnati Children’s Hospital Medical Center

## Abstract

Fragile X Syndrome (FXS) is an X-linked disorder leading to the loss of expression of *FMR1*-protein product, FMRP. The absence or deficiency of FMRP is thought to result in the characteristic FXS phenotypes, including intellectual disability. Identifying the relationship between FMRP levels and IQ may be critical to better understand underlying mechanisms and advance treatment development and planning. A sample of 80 individuals with FXS (67% male), aged 8–45 years, completed IQ testing and blood draw via venipuncture to determine the relationship between IQ scores and FMRP levels as well as the normalcy of IQ distributions. In females with FXS only, higher FMRP levels were associated with higher IQ. In contrast, males with FXS showed a downward shifted but otherwise normal distribution of IQ scores. Our findings offer a paradigm-shifting views of FXS—males with FXS have normally distributed IQ that is downshifted 5 standard deviations. Our novel work provides evidence of a “FXS standard curve”, and is a critical step towards establishing molecular markers of disease severity in FXS. There is much future work to better understand the mechanism by which FMRP loss leads to intellectual disability and what biological/genetic and socio-environmental factors contribute to variation in IQ.

## Introduction

The Fragile X messenger ribonucleotide 1 (*FMR1*) gene-specific protein product, fragile X messenger ribonucleotide protein (FMRP), is critical for normal brain development based on its role in synaptogenesis, especially within the cerebral cortex, cerebellum, and hippocampus, and in modifying synaptic structure in response to environmental stimulation. Unstable mutation of *FMR1* located on the long arm of the X chromosome involves the expansion of trinucleotide CGG repeats in the promotor region of the gene, with large expansions of > 200 CGG repeats considered to be “full mutations” resulting in at least partial gene methylation and deficient or absent FMRP expression. The absence of FMRP is thought to result in the clinical phenotype of full mutation FXS in males (FXS(M) marked by moderate to severe intellectual disability, facial dysmorphia, anxiety, and hyperactivity. A milder presentation of these clinical phenotypes is observed in females with FXS (FXS(F)), due to random X-inactivation patterns and associated significant variation in *FMR1* mRNA and FMRP expression^1–3^, and in a subset of males with FXS due to size or methylation mosaicism (Mosaic(M)^4^. Numerous studies over the past 20 years have demonstrated that reduction in FMRP levels are associated with a greater degree of cognitive impairment among males and females with full mutation FXS^5–16^. Degradation in performance is observed across cognitive domains, but especially in areas of processing speed, working memory, cognitive flexibility, and inhibitory control. However, there are several limitations of these past studies. For example, previous studies were largely limited to Mosaic(M) and FXS(F). In addition, floor effects on standardized IQ measures and low sensitivity of blood sample assays to extremely low levels of FMRP has limited the ability to examine whether FMRP expression and IQ correlate among FXS(M) who tend to have no protein expression.

Our group recently optimized and validated a highly sensitive and reproducible FMRP assay that detects FMRP expression at extremely low values^1^. In fact, we found that 30–40% of FXS(M), who based on standard PCR and Southern Blot testing are considered full mutation, fully methylated, express trace levels of FMRP in their blood. This is consistent with findings of incomplete silencing of the FMR1 gene reported by our group and others^2,17^. In each of our recent papers, we reported a small to medium sized correlation between Deviation IQ^18^ and FMRP and mRNA levels^1,2^. However, when examining FXS(M) alone, these relationships was no longer significant. Based on visual inspection of scatterplots, it is evident that there is a wide range of IQ scores among FXS(M) in the original samples (Z deviation IQ range − 20 to 47) that corresponded to no to very low protein expression. Thus, the current study aimed to re-examine the distribution of IQ scores in a larger sample of individual with FXS and explore the relationship with FMRP levels. Establishing how FMRP relates to cognitive variation in FXS, especially among FXS(M), is critical to understanding the neurobiological sequelae whereby the absence or deficiency of FMRP leads to intellectual disability.

## Results

**Figure 1:**
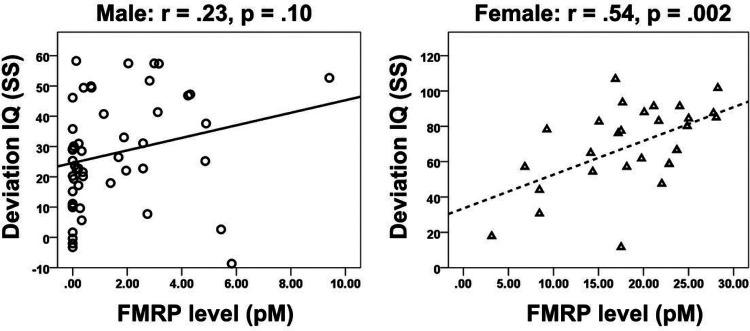
Linear relationships between FMRP levels and Deviation IQ in Males and Females with FXS. (Left) Males with FXS (n=51), as depicted by open circles, do not demonstrate a significant relationship between Deviation IQ and FMRP levels. (Right) Females with FXS (29), as depicted by open triangles, demonstrate a significant relationship between Deviation IQ and FMRP levels. *FMRP* Fragile X Messenger Ribonucleoprotein, *FXS* Fragile X Syndrome, *pM* picomolar, *IQ* Intelligence Quotient, *SS* Standard Score

Among all males with FXS, FMRP level was not significantly related to IQ Deviation score (r = .23, p = .10; Fig. 1A). Visually examination of the scatterplot reveals a wide distribution of IQ scores for males with no protein expression or very low protein expression. However, individual data points tend to fit the regression line more closely at higher protein expression values (≥ 3 pM). In contrast, among females with FXS, FMRP level was significantly and positively related to IQ Deviation score (r = .54, p = .002; Fig. 1B) with higher levels of FMRP being related to higher IQ scores. Visual examination of the scatterplot confirms a tighter relationship between FMRP and IQ scores in females as compared to in males.

**Figure 2:**
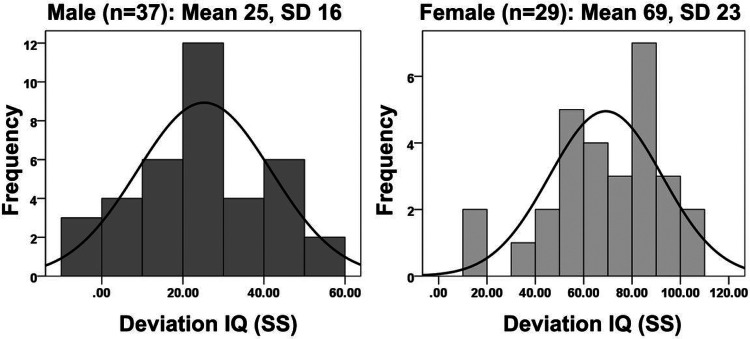
Distribution of Devation IQs in Males (Dark Gray) and Females (Light Gray) with FXS with Normal Distribution Curve Overlayed. (Left) A subset of males with FXS who express < 2.5pM FMRP (n=37) demonstrated a near-normal distribution of Deviation IQ scores that is downshifted five standard deviations. (Right) Females with FXS (n=29) also shows evidence of a relative normal distribution of Deviation IQ scores that is downshifted two standard deviations.

Examining the distribution of IQ scores for males with low FMRP (< 2.5 pM; n = 25), we found they had a mean IQ of 25 and standard deviation of 16 with skewness and kurtosis values of 0.3 and − 0.5, respectively (Fig. 2A). Examining the distribution of IQ scores for females, we found they had a mean IQ of 69 and standard deviation of 23 with skewness and kurtosis values of −0.8 and 0.4, respectively (Fig. 2B). Although skewness and kurtosis values for males and females are relatively similar and indicate near symmetry and normalcy, visual examination of the histograms demonstrates slight leftward skewing of female IQ consistent with a negative skewness value.

## Discussion

With our lab’s recent optimized FMRP assay that can detect levels at very low values^1^, it has become paramount to establish the clinical utility of this highly quantitative continuous measure. We replicate ours and others previous findings that FMRP expression and IQ *are not* related in males with FXS^10^, but are related in females with FXS^10,11,19^. However, when examining the distribution of IQ scores in males with no or low FMRP expression, we found a remarkably near-normal distribution. Hessl and colleagues^20^ reported that normalized cognitive scores “exhibited a more ‘normal’ distribution”, but ours is the first study that has specifically examined the skewness and kurtosis of the distribution of IQ scores in males with FXS whose FMRP expression is known.

We found that full mutation, fully methylated males with FXS demonstrated a near normal distribution of Deviation IQ scores. The mean Deviation IQ for this subgroup was shifted approximately 5 standard deviations (SDs) below the population mean of 100, but with a SD of 15 and acceptable kurtosis and skewness values it still demonstrated a normal distribution. This is the first time, to the best of our knowledge, this has been systematically examined in FXS, and specifically within this subgroup of males.

Our current finding of a downward shifted, but otherwise normal distribution of IQ scores in FXS, previously has been observed in multiple other genetic syndromes associated with intellectual disability (ID) including Prader-Willi Syndrome^21^, Williams Syndrome^22^, Velo-Cardio-Facial-Syndrome^23^, and Tuberous Sclerosis^24^. In Down Syndrome, IQ scores demonstrate negative skew, but mental-age (MA) scores demonstrate near-normal distribution^25^. Thus, each of these genetic syndromes seemingly have their own “standard curve” with their own mean and SD of IQ scores. Thus, in conjunction our findings emphasize the global effect of the disorder-specific genes on IQ. Within context of FXS, we propose that one effect of the absence of FMRP in FXS(M) is to shift the cognitive ability distribution downwards by about 75 IQ points (or 5 SDs).

It largely remains unknown in FXS as well as in these other syndromic IDs what accounts of the remaining ‘normal’ variance in IQ. We suspect factors known to impact IQ in typically-developing children including maternal IQ, parental education, SES, and and/or social-environment and health factors (i.e., nutrition, growth) would similarly impact IQ in FXS and other syndromic IDs. Only a few studies have explored whether these factors account for variance in IQ in syndromic IDs. For example, De Smedt and colleagues identified a positive effect of parental educational attainment level on FSIQ of individuals with VFC^23^. In FXS, Dyer-Friedman and colleagues found that both biological/genetic factors (i.e., parental IQ score) as well as social-environmental factors (i.e., parental support for learning and enrichment) accounted for variance in cognitive function in males and females with FXS^13^. Of note, biological/genetic and social-environmental factors affected specific aspects of cognition somewhat differently for males and females with FXS. Specifically, parental IQ broadly accounted for variance across cognitive domains in females, whereas parental IQ only impacted areas of fluid intelligence like processing speed and perceptual organization in males. In contrast, a more enriching home environment was related not only to overall cognitive development in males and females with FXS but also verbal and attention skills. These findings implicate a complex mechanism in which a combination of biological/genetic and social-environmental factors *in addition to FMRP* impact cognitive outcomes in FXS. Thus, future studies are needed to best understand facilitators and risk factors for optimal cognitive outcomes in FXS.

FMRP levels at higher values and/or within mosaic males and females with FXS may on their own be better able to predict intellectual ability. However, in the absence or near absence of FMRP, we propose that the approach to predicting ability level shifts to examining IQ relative to other FXS(M). Our findings in conjunction with those discussed above, suggest that within each syndromic ID, we would expect syndrome-specific standard, or “normal”, curves, with individual profiles within that standard curve as a result of individual genotypic and environmental influences. Although our study shows the most normalcy of IQ scores within FXS(M), it is possible that with a larger dataset we also will see a more normal distribution of IQ scores in FXS(F). A larger sample also would allow us to determine whether FXS males with mosaicism have a normal distribution of IQ scores.

We propose one way to conceptualize these “syndromic standard curves” is to think of them as ability distributions. This can help parents and providers identify where a child with syndromic ID falls on that specific syndrome’s standard curve. In other words, where does the individual’s IQ score fall relative to other individuals with that same syndromic ID? Typically, IQ scores of individuals with syndromic ID are presented relative to typically-developing controls, which only provides minimum information of ability and impairment given the majority of syndromic IDs fall within the moderate to severe intellectual disability range. One can image the immense benefit to families and providers to be able to communicate cognitive ability in this “new” way. Providers with a caseload of a specific syndrome will be able to help caregivers more effectively discuss short- and long-term treatment planning when IQ is thought of in this lens.

For example, think about a male patient with FXS with a Deviation IQ of 10. Based on our findings, this score falls 1 SD below the “FXS” mean. In contrast, think about a male patient with FXS with an IQ of 40 is 1 SD above the “FXS” mean. Ability level, expectations for academic achievement and functional daily living skills, and planning for adulthood would look different for those two patients based where they fall on the “FXS” standard curve. Thus, replicating and extending findings with a much larger sample of individuals with FXS is critical to being able to put these standard curves into clinical practice.

It is important to note our current sample overwhelmingly identifies as White, non-Hispanic, and thus our “standard curve” may be biased towards this population and not adequate represent individuals with FXS from under-represented, minority populations. In addition, we did not collect parental educational attainment or socioeconomic information from participants, furthering limiting the ability to generalize our findings. Examining these social-environmental factors as well as other biological factors contributing to this variation in cognitive ability is outside the scope of the current study, but remains an essential future direction. Further, familial studies are needed to estimate the heritability of intellectual ability in the FXS population and determine the relative contribution of biological/genetic versus social-environmental factors to variability within the distribution. Last, future studies should determine whether similar “standard curves” exist for other clinically-relevant factors, including adaptive skills.

In conclusion, among males with near or complete absence of FMRP, we provide novel evidence that IQ is downshifted 5 standard deviations, but is otherwise relatively “normally distributed” indicating FMRP is responsible for this downward shift, but does not account for the remaining variance in cognition. This is in contrast to ours and others’ findings indicating FMRP has a dose-dependent effect on IQ in mosaic males and full mutation females who express higher levels of protein. There is much future work to better understand the mechanism by which deficient or absent FMRP leads to clinical phenotype and what biological/genetic and/or socio-environmental factors contribute to variation in IQ scores among these males. Still our novel work is a critical step towards establishing molecular markers of disease severity in FXS and has critical clinical implications for treatment and care planning, especially for full mutation males with FXS.

## Methods

### Participant Sample

Participants were recruited through the Cincinnati Fragile X Research and Treatment Center and were only included in the current study if they completed the two primary measures of interest—blood collection for FMRP processing and the Stanford Binet-5 (SB-5). A total of 80 individuals diagnosed with FXS (n = 51, 64% males) ages 8–45 years old completed testing (Table 1). Among the 51 males, 35 were classified as full mutation, fully-methylated, 11 as size mosaics and 3 as methylation mosaics based on standard PCR and Southern Blot testing completed by Elizabeth Berry-Kravis’s lab at Rush University. Two males had “unknown” mosaic status due to completed PCR, but not Southern Blot not yet completed to confirm methylation status.

### Blood Collection, Processing, and Luminex-Based FMRP Blood Quantification

Blood samples were collected in 2 mL Vacutainer K2EDTA tubes and inverted 10 times before processing to ensure homogeneity within the sample. 50 uL of blood was pipetted onto ID Bloodstain Cards (Whattman) producing two cards with 13 spots each from one sample collection. Cards were dried and stored with desiccant packs in low-gas-permeable bags to ensure DBS stability within 4–24 hours after spotting. Full details describing blood processing and the Luminex-based FMRP quantification technique previously have been described^1^. This methodology has established test-retest reproducibility and in doing so showed the ability to consistently discriminate between zero and trace levels of FMRP in blood.

### Assessment of Intellectual Functioning

Participants completed the Abbreviated Battery of the Stanford-Binet, Fifth Edition (SB-5). Standard scores were converted to Deviation IQ scores and scaled scores were converted to z-scores in order to reduce floor effects present for individuals with severe cognitive impairments, and thus to better estimate intellectual ability and capture inter-individual variability^18^.

### Statistical Analysis

All statistical analyses were completed in SPSS. Pearson correlations between FMRP levels and Deviation IQ scores were conducted separately for males and females with FXS. Based on our previous findings of a lack of correlation between FMRP and IQ in FXS(M) and the wide range of IQ scores for males with no to low protein levels, we examined the distribution of IQ for males and females with FXS separately. In order to restrict our male sample to those with most similar genotype, while also maximizing our sample number, we only included males in our skewness and kurtosis analyses with FMRP < 2.5pM regardless of mosaic status.

Skewness is a measure of asymmetry, such that values of 0 indicate perfect symmetry, negative values indicate leftward skewed data (i.e., longer left tail than right), and positive values indicate rightward skewed data. In other words, a normal distribution has a skewness of 0 and near-normal distribution should have values close to 0. On the other hand, kurtosis is a measure of how “heavy” tailed the data is relative to a normal distribution. Kurtosis value of 0 indicates a normal distribution, and a positive (and high) kurtosis value reflects a “heavy tailed” distribution or data with many outliers, whereas a negative kurtosis value indicates a “light tailed” distribution that is relatively uniform or flat. By convention, skewness and kurtosis values between − 0.5 and 0.5 indicate data are nearly symmetrical, normal distribution.

## Figures and Tables

**Table 1:** Values given as Mean (Standard Deviation), Range;

	FXS Male n = 51	FXS Female n = 29
**Age**	24.7 (10.3) *8–45*	21.4 (9.5) *8–42*
**Deviation IQ**	27.6 (18.1)* −8.7–58.3	69.1 (23.4) *11.6–106.9*
**Non-verbal Z-score**	−5.4 (1.7)* −9.1–1.7	−2.5 (1.9) *−7.2–0.4*
**Verbal Z-Score**	−4.2 (1.2)* −7.3–1.7	−1.8 (1.5) *−4.6–0.7*
**FMRP**	1.5 (2.0)* 0–9.4	18.6 (6.6) *3.1–28.3*

* p < 0.001

For reference, FMRP in typically-developing controls is 28.2 (5.8), *18–43*

## Data Availability

The dataset used and/or analyzed during the current study are available from the corresponding author on reasonable request.
